# New bone formation accelerates during lower limb lengthening and deformity correction in children with Ollier’s disease

**DOI:** 10.1186/s10195-023-00717-3

**Published:** 2023-07-31

**Authors:** Chunxing Wu, Yiyong Huang, Peng Huang, Yueqiang Mo, Dahui Wang, Bo Ning

**Affiliations:** 1 Department of Pediatric Orthopaedics, Children’s Hospital of Fudan University, National Children’s Medical Center, 201102 Shanghai, China; 2 Department of Pediatric Orthopaedics, Hainan Women and Children’s Medical Center, Haikou, 570206 China

**Keywords:** Ollier’s disease, Osteotomy, Lower limb lengthening, External fixation, Bone healing index, New bone formation

## Abstract

**Background:**

Ollier’s disease can cause severe length discrepancy of the lower extremities and deformity in children. Osteotomy and limb lengthening with external fixation can correct the limb deformity. This study evaluated (1) whether the duration of external fixation was reduced in patients with Ollier’s disease, and (2) the incidence of complications such as pin tract infection, external fixation loosening, and joint stiffness.

**Methods:**

Two groups were compared with respect to age, angular correction (AC), lengthening gap (LG), distraction index (DI), lengthening length (LL), lengthening length percentage (L%), lengthening index (LI), bone healing index (BHI), and external fixation index (EFI). Group 1 (Ollier’s disease) comprised nine patients undergoing 11 lower limb lengthening procedures using external fixators; group 2 (control, normal lengthened bone) comprised 28 patients undergoing 29 lengthening procedures with external fixators.

**Results:**

In patients with Ollier’s disease, full correction of the deformity and full restoration of length were achieved in all cases. In the femur, the mean AC (15.97° vs. 6.72°) and DI (1.11 mm/day vs. 0.78 mm/day) were significantly larger, while the LI (9.71 days/cm vs. 13.49 days/cm), BHI (27.00 days/cm vs. 42.09 days/cm), and EFI (37.86 days/cm vs. 56.97 days/cm) were all significantly shorter in group 1 than in group 2 (*p* < 0.05). In the tibia, the mean AC and L% were larger, while the LG, LI, BHI, and EFI were all shorter in group 1 than in group 2. There was no significant difference between the two groups in the incidence of complications.

**Conclusion:**

In children with Ollier’s disease, new bone formation accelerated and the healing speed of the lengthened segments was faster throughout the whole lengthening period with external fixation, and full correction of the deformity and full restoration of length could be achieved.

*Level of Evidence* III

## Introduction

Ollier’s disease, originally described in 1899 and also known as multiple enchondromatosis, is a rare nonhereditary skeletal disorder [1] with an estimated prevalence of 1:100,000 [2]. Although the inheritance patterns for Ollier’s disease are unclear, mutations in two genes (PTHR1 and IDH1) are thought to be responsible [3–5].

This disease is attributable to a failure of normal endochondral ossification, and results in various problems linked to bowing of the long bones, longitudinal growth deformities such as genu varus, broadening of the metaphyses, and limb length discrepancy (LLD) [6–11]. If untreated, LLD can reach as much as 10–25 cm at maturity [12]. There is no effective treatment for Ollier’s disease itself; complete curettage of the enchondromas is realistically impossible, as the lesions are extensive [7].

For the last few years, external fixation using systems including mono-lateral fixation (such as the OrthoFix fixation systems) and circular fixation (such as the Ilizarov fixation or Taylor spatial frame, TSF) have become popular treatment options for the correction of LLD and angular deformity to improve the patient’s quality of life.

In this study, we compared patients who underwent leg lengthening to treat Ollier’s disease with others who underwent leg lengthening for other reasons. We also addressed some questions regarding treatment for Ollier’s disease as follows: (1) what is the optimal duration of the external fixation, and (2) what is the incidence of complications such as pin tract infection, external fixation loosening, and joint stiffness?

## Patients and methods

Group 1 (Ollier’s disease) consisted of nine patients (four boys and five girls) who underwent 11 progressive limb lengthening procedures performed using an Ilizarov (two cases), a TSF (four cases), or an OrthoFix (five cases) fixation between 2018 and 2020. The mean age of the patients at operation was 7.3 years (range 3.1 to 14.0 years). Monosegmental lengthening involved the femur in seven cases and the tibia in four. One patient underwent two lengthening procedures in the same tibia, and one patient underwent two lengthening procedures, one in the femur and one in the tibia.

Group 2 (control, lengthened segments normal) consisted of 28 patients and involved 29 lengthening procedures using an Ilizarov (11 cases), a TSF (five cases), or an OrthoFix (13 cases) fixation for monosegmental lengthening between 2017 and 2021. The mean age of these patients at operation was 9.3 years (range 3.1 to 14.2 years). The lengthening was required as a result of six types of disorders (congenital limb length discrepancy and deformity with normal bone: 15 cases; residual deformity after fracture: seven cases; residual deformity after osteomyelitis: four cases, in which the healthy bone segments were lengthened, avoiding the segment with osteomyelitis; congenital femoral dysplasia: one case; hemilateral dysplasia: one case; Legg–Calve–Perthes disease: one case) and were unilateral in all 28 patients. The lengthened segments were healthy, with no lesions. The procedures were performed 18 times for the femur and 11 times for the tibia.

## Operative technique

All deformities were evaluated using the center of rotation and angulation (CORA) method. All osteotomies were performed at the CORA level, and wires and half pins were inserted into the segments according to the osteotomy position, not considering intralesionally.

One week after the osteotomy, each patient was examined by X-ray, lengthening of the segment was begun, and X-rays were taken every 2 weeks thereafter. The number of days from osteotomy to the start of lengthening was called the lengthening gap (LG). When the target length and angular correction were achieved, the segment extension was stopped and an X-ray was taken every 1 month until the lengthened bone was healing well. We considered consolidation to be completed when anteroposterior and lateral radiographs found that three of four cortices of the regenerated bone in the distraction gap were bridged [10].

After the removal of the external fixator, according to age and the results of an X-ray examination, the lengthened segment was secured by a removable semicircular splint for a 2- to 6-week period. Full weight-bearing was initiated after splint removal.

**Fig. 1:**
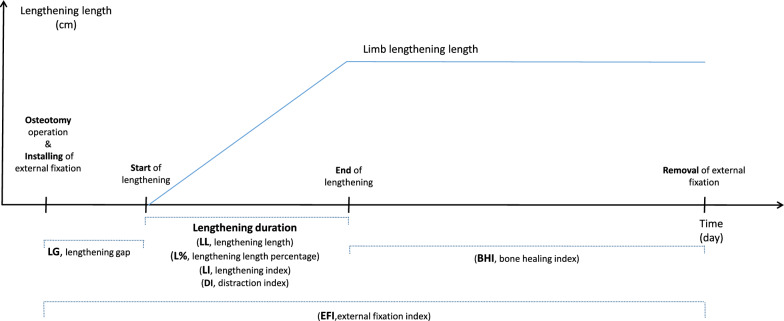
Objective outcomes according to lengthening duration

The clinical results were evaluated based on objective outcomes, as shown in Table 1 and Fig. 1.

**Table 1:** Objective outcomes and explanation

Name	Abbreviation	Explanation
Angular correction (°)	AC	The amount of correction of angular deviation (°)
Lengthening gap (days)	LG	The number of days from osteotomy to the start of lengthening (days)
Distraction index (mm/day)	DI	The amount of lengthening per day (mm/day)
Lengthening length (cm)	LL	The total amount of lengthening (cm)
Lengthening length percentage (%)	L%	The lengthening length as a percentage of the initial length (%)
Lengthening index (days/cm)	LI	The duration of external fixation lengthening in days divided by the amount of lengthening in cm (days/cm)
Bone healing index (days/cm)	BHI	The time until bony union in days in the frame divided by the amount of lengthening in cm (days/cm)
External fixation index (days/cm)	EFI	The total time in the external fixation in days divided by the amount of lengthening in cm (days/cm)

## Statistical analysis

Objective outcomes (LG, DG, LL, L%, LI, BHI, and EFI) were compared using Student’s *t*-test for independent samples and the chi-square test. The level of significance was set to ^*^p < 0.05, ^**^p < 0.01. The descriptive statistical values included the mean and standard deviation. Analysis was performed using SPSS 17 software (SPSS Inc., Chicago, IL, USA).

## Results

**Fig. 2:**
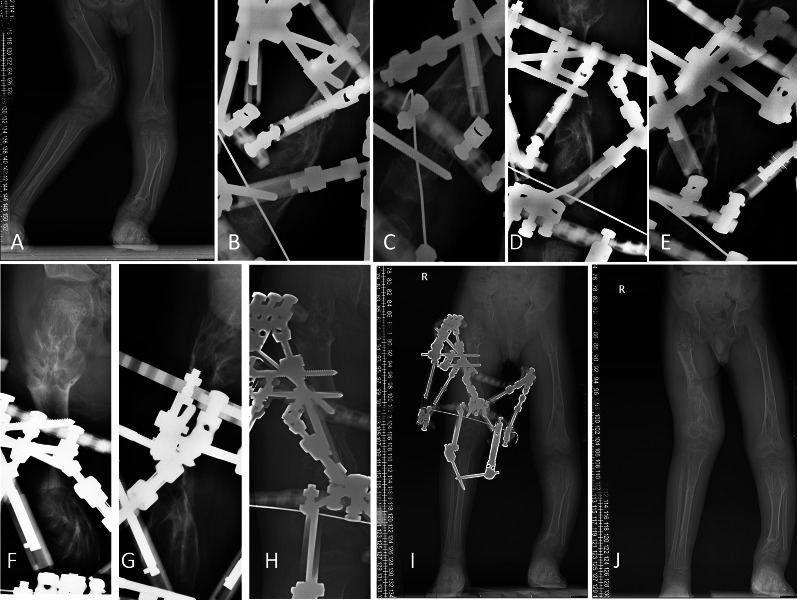
A 5Y1M boy (patient 9) with Ollier’s disease, right femur lengthened by 3 cm, 33°AC, with TSF fixator. **A** AP radiograph before operation. **B**, **C** AP and lateral radiographs during operation. **D**, **E** AP and lateral radiographs after 7 days of lengthening. **F**, **G** AP and lateral radiographs after 26 days of lengthening. **H** AP at the end of lengthening after 32 days. **I** AP before external fixation removal, 3.5 months after operation. **J** Full length AP 1 month after removal of the external fixator

**Fig. 3:**
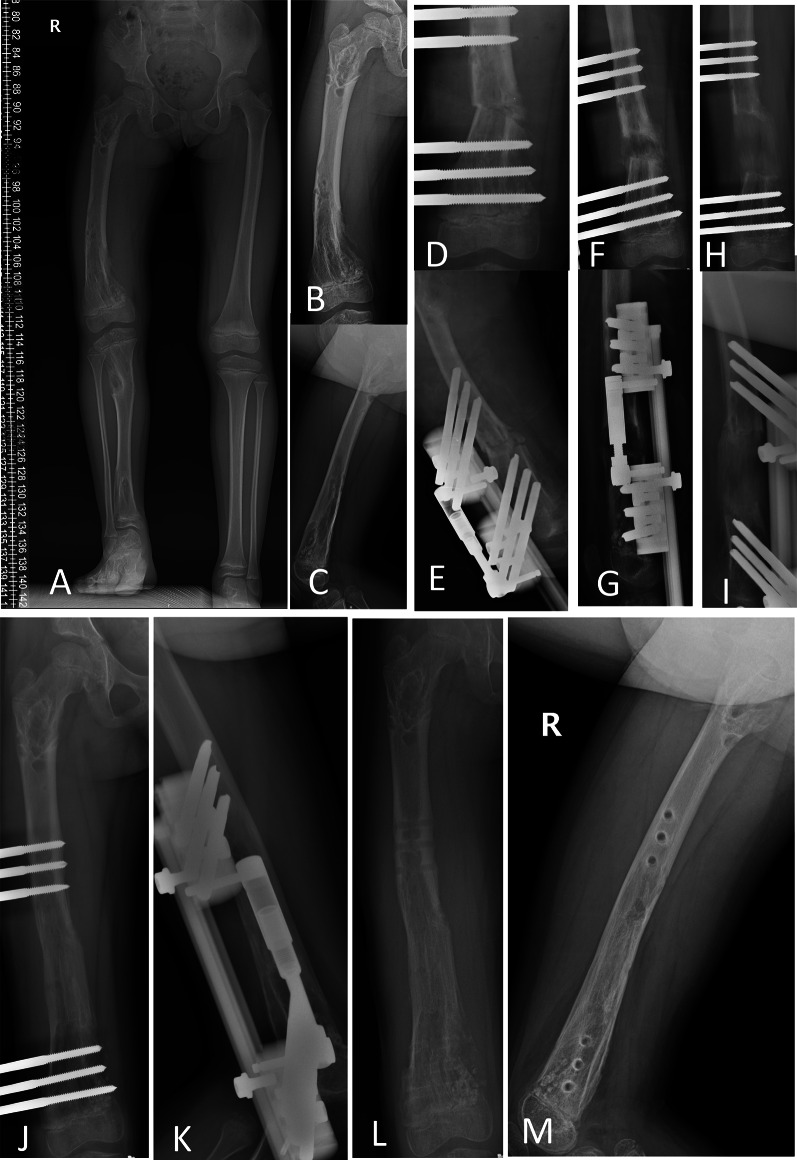
A 4Y10M girl (patient 2) with Ollier’s disease, right femur lengthened by 5.90 cm, 16.4°AC, with OrthoFix fixator. **A** Preoperative full-length AP. **B**, **C** AP and lateral radiographs before operation. **D**, **E** AP and lateral radiographs during operation. **F**, **G** AP and lateral radiographs after 30 days of lengthening. **H**, **I** AP and lateral radiographs at the end of lengthening after 61 days. **J**, **K** AP and lateral radiograph before external fixation removal, 6 months after operation. **L**, **M** AP and lateral radiographs 1 month after removal of the external fixator

**Fig. 4:**
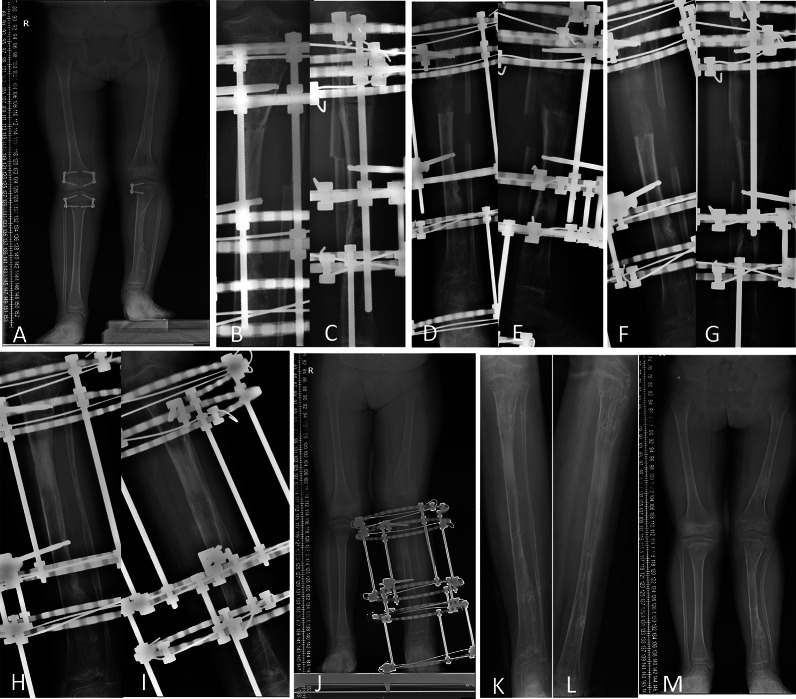
An 8Y11M girl (patient 6) with Ollier’s disease, left tibia lengthened by 6.65 cm, 6.4°AC, with Illizarov fixator. **A** Preoperative full length AP radiograph. **B**, **C**, AP and lateral radiographs during operation. **D**, **E** AP and lateral radiographs after 38 days of lengthening. **F**, **G** AP and lateral radiographs at the end of lengthening after 68 days. **H**, **I**, AP and lateral radiographs before removal of the external fixator, 7.5 months after operation. **J** Full-length AP radiograph before removal of the external fixator, 8 months after operation. **K**, **L** AP and lateral radiographs after removal of the external fixator, 8 months after operation. **M** Full-length AP radiograph 6 months after removal of the external fixator

Characteristics of patients with Ollier’s disease (group 1) are shown in Table 2 and in Figs. 2, 3, and 4.

**Table 2:** Characteristics of patients with Ollier’s disease (group 1)

Patient	Case	Gender	Age	Segment	Method	LG (days)	AC (°)	Angular deviation	DI (mm/day)	LL (cm)	L% (%)	LI (days/cm)	BHI (days/cm)	EFI (days/cm)
1	1	Boy	5Y2M	Left femur	OrthoFix	8	12.4	Varus	0.94	5.00	20.70	10.60	19.60	31.80
2	2	Girl	4Y10M	Right femur	OrthoFix	5	16.4	Varus	0.88	5.90	22.05	11.36	14.24	26.44
3	3	Girl	3Y1M	Left femur	OrthoFix	5	22.0	Varus	1.18	5.42	26.11	8.49	19.37	28.78
4	4	Boy	14Y	Right femur	TSF	7	20.0	Valgus	1.97	6.30	15.79	5.08	36.67	42.22
5	5	Girl	4Y9M	Left femur	OrthoFix	5	8.0	Varus	0.83	5.00	23.36	12.00	26.60	39.60
6	6	Girl	8Y11M	Left tibia	Ilizarov	8	6.4	Varus	1.02	6.65	29.08	9.77	25.26	36.09
7	7	Girl	7Y2M	Left temur	OrthoFix	7	0	/	1.02	5.53	18.94	9.76	46.84	57.87
8	8	Boy	9Y6M	Right tibia	Ilizarov	7	4.80	Valgus	0.75	3.24	13.36	13.27	25.93	41.36
	9	Boy	11Y6M	Right tibia	TSF	6	25.0	Anteverted	0.74	4.80	15.19	13.54	40.83	55.63
9	10	Boy	5Y1M	Right femur	TSF	6	33.0	Valgus	0.94	3.00	12.21	10.67	25.67	38.33
	11	Boy	5Y10M	Left tibia	TSF	5	9.3	Varus	0.59	5.50	29.26	16.87	38.00	63.27

In group 1 (Ollier’s disease), the follow-up period ranged from 18.3 to 52.3 months (mean 30.1 months). In group 2 (control), the follow-up period ranged from 22.4 to 65.5 (41.1) months.

### Comparison between the two groups

In the femur, the mean AC (15.97° vs. 6.72°, *p* = 0.022*) and DI (1.11 mm/days vs. 0.78 mm/days,* p* = 0.006^**^) were significantly larger in group 1 than in group 2, while LI (9.71 days/cm vs. 13.49 days/cm, *p* = 0.020^*^), BHI (27.00 days/cm vs. 42.09 days/cm, *p* = 0.019^*^), and EFI (37.86 days/cm vs. 56.97 days/cm, *p *= 0.007^**^) were all significantly shorter in group 1 than in group 2, and age at operation (6.30 years vs. 9.92 years, *p* = 0.032^*^) was significantly younger in group 1 than in group 2.

In the lower extremity, the mean AC (14.30° vs. 5.90°, *p* = 0.011^*^) and DI (0.99 mm/days vs. 0.80 mm/days, *p* = 0.049^*^) were significantly larger in group 1 than in group 2, while LG (6.27 days vs. 7.17 days, *p* = 0.033^*^), BHI (20.00 days/cm vs. 41.24 days/cm, *p* = 0.011^*^), and EFI (41.94 days/cm vs. 56.26 days/cm, *p* = 0.009^**^) were all significantly shorter in group 1 than in group 2.

When considering the tibia, femur and lower extremity together, the mean AC, L%, and DI were all larger in group 1 than in group 2 (except that DI was similar in the tibia), while LG, LI, BHI, and EFI were all shorter in group 1 than in group 2.

The details of group 1 and group 2 are shown in Table 3.

**Table 3:** Objective outcomes in both groups

		Age (year)	AC (°)	LG (days)	DI (mm/day)	LL (cm)	L% (%)	LI (days/cm)	BHI (days/cm)	EFI (days/cm)
Femur	Group 1(*N* = 7)	6.30 ± 3.60*	15.97 ± 10.60*	6.14 ± 1.22	1.11 ± 0.40* *	5.17 ± 1.06	19.88 ± 4.70	9.71 ± 2.33*	27.00 ± 11.29*	37.86 ± 10.59**
	Group 2 (*N* = 18)	9.92 ± 3.54*	6.72 ± 7.49*	7.06 ± 1.06	0.78 ± 0.16**	5.41 ± 1.22	16.06 ± 4.98	13.49 ± 3.69*	42.09 ± 14.15*	56.97 ± 15.56**
	*p*	0.032*	0.022*	0.075	0.006**	0.648	0.094	0.020*	0.019*	0.007**
Tibia	Group 1 (*N* = 4)	8.92 ± 2.35	11.38 ± 9.27	6.50 ± 1.29	0.78 ± 0.18	5.05 ± 1.43	21.72 ± 8.63	13.36 ± 2.90	32.50 ± 8.07	49.09 ± 12.56
	Group 2 (*N* = 11)	7.77 ± 3.42	4.55 ± 12.17	7.36 ± 1.29	0.83 ± 0.29	5.23 ± 1.05	19.89 ± 4.68	13.78 ± 5.59	39.84 ± 13.70	55.08 ± 16.19
	*p*	0.551	0.261	0.271	0.755	0.787	0.600	0.891	0.256	0.517
Lower extremity (femur + tibia)	Group 1 (*N* = 11)	7.26 ± 3.35	14.30 ± 9.93*	6.27 ± 1.19*	0.99 ± 0.36*	5.12 ± 1.14	20.55 ± 6.04	11.04 ± 3.03	20.00 ± 10.18*	41.94 ± 12.10**
	Group 2 (*N* = 29)	9.28 ± 3.63	5.90 ± 8.50*	7.17 ± 1.14*	0.80 ± 0.22*	5.34 ± 1.14	17.52 ± 5.14	13.60 ± 4.41	41.24 ± 13.78*	56.26 ± 15.54**
	*p*	0.116	0.011*	0.033*	0.049*	0.591	0.120	0.085	0.011*	0.009**

*LG* lengthening gap, *DI* distraction index, *LL* lengthening length, *L%* lengthening length percentage, *LI *lengthening index, *BHI* bone healing index, *EFI* external fixation index

The difference was significant: *p < 0.05, **p < 0.01

In group 1, knee range of motion recovery was obtained within 0.5 to 9 months after removal of the external fixator. The reported complications were: one case of limited knee movement (1/11, 9.1%), one case of pin tract infection (1/11, 9.1%), one case of pathological fracture (pathological fracture in the proximal femur while the osteotomy position was in the distal femur) (1/11, 9.1%), and one case of early consolidation (1/11, 9.1%). In group 2, the knee recovered its normal range of motion 1 to 18 months after fixation removal. The reported complications were: two cases of limited knee movement (2/29, 6.9%), one case of pin tract infection (1/29, 3.4%), and two cases of pathological fracture (2/29, 6.9%) after external fixation removal. In both group 1 and group 2, no complication such as vascular impairment, neurapraxia, or non-union was observed during the lengthening or after fixation removal.

There was no significant difference between group 1 and group 2 in the incidence of complications such as joint stiffness, infection, pathological fracture, early consolidation, vascular impairment, neurapraxia, or non-union (Table 4).

**Table 4:** Complications in the lengthened segments in patients with and without Ollier’s disease

Complication	Group 1 (Ollier’s disease) case, %, (*N* = 11)	Group 2 (control) case, %, (*N* = 29)	*P * (chi-square test)
Joint stiffness	1, 9.1%	2, 6.9%	0.814
Infection	1, 9.1%	1, 3.4%	0.465
Pathological fracture	1, 9.1%	2, 6.9%	0.814
Early consolidation	1, 9.1%	0, 0	0.100
Vascular impairment	0	0	/
Neurapraxia	0	0	/
Non-union	0	0	/

## Discussion

The manifestations of Ollier’s disease usually appear in early childhood because multiple enchondromas near the epiphyseal plate cause severe progressive growth inhibition and angular deformity. As a result of erosion of the adjacent physis, tethering of the physis by a bridging tumor, or abnormally thick periosteal sleeves formed in reaction to a tumor, the diaphyses of the affected bones seem short and widened [13]. Fractures through the tumors may result in induced deformity and LLD [2].

Traditional treatments for Ollier’s disease are similar to those for other benign bone lesions, including enchondromas, and include excision by curettage, corrective osteotomies, bone grafting, and internal fixation [14]. However, complete curettage of the enchondromas is realistically impossible because the lesions are extensive [7]. These methods also cannot resolve the severe LLD.

Recently, Huser et al. reported successful lower limb lengthening using implantable lengthening nails for patients with Ollier’s disease (six patients, 11 segments lengthened) if the bone size and morphology permits. Gradual correction can only be used for length, not rotation or alignment. Acute rotation and deformity correction can only be performed during surgery [15]. However, external fixators allow gradual correction in all planes and correction of LLD, although the risk of transcutaneous fixation infection remains, and the bulk of the fixator presents a major nuisance for patients.

With the development of external fixation technology, the bowing and shortening of a bone segment can often be corrected by corticotomy and lengthening. Some researchers have expressed concerns that the affected bone may be weakened by Ollier’s disease and consequently treatment may result in pathological fractures [8–10]. However, other researchers have suggested that lengthening is no more complex compared to cases of LLD due to other causes aside from Ollier’s disease because the growth disorder only involves bone; the soft tissues are normal [16, 16]. In our study, there was no difference in the incidence of complications in the lengthened segments in patients with and without Ollier’s disease when joint stiffness, infection, pathological fracture, vascular impairment, neurapraxia, and non-union were evaluated. External fixation provided sufficient stability for deformity correction and limb lengthening.

Some doctors have noticed that a shorter healing time and early consolidation of the lengthened segment are more common in some patients with Ollier’s disease [18]. There are two solutions to deal with the early consolidation. One is to increase the lengthening rate; the other is to perform osteotomy repeatedly to continue lengthening. Myers noticed a tendency for hypertrophic bone regeneration in patients with Ollier’s disease [19] and recommended a higher rate of distraction. Madan [9] noticed three cases of early consolidation in ten patients that required manipulation under anesthesia and osteoclasis or cessation of lengthening. The target lengthening length can be achieved by performing repeated corticotomy and repeated lengthening. However, this method increases the number of operations, the pain experienced by the patient, and the economic burden. Our preferred option is to increase the lengthening rate. There may be a higher risk of infection, delayed healing, or nonunion, so an appropriate distraction rate that can achieve the target extension length with normal bone union, preventing delayed bone union and nonunion and reducing the hardness of the lengthened segment, should be chosen to meet these demands.

There are different and even contradictory data regarding BHI, EFI, and other measures in different studies. Reported femoral BHI ranged from 22.5 to 35 days/cm, tibial BHI ranged from 21 to 35.7 days/cm, and polysegmental femorotibial BHI ranged from 19.9 to 31.8 days/cm [17, 20–23]. Watanabe et al. [10] reported lower limb segment EFI values of between 39.7 days/cm in an intralesional distraction osteogenesis group and 30.8 days/cm in an extralesional distraction osteogenesis group. Some of these differences could be caused by the fact that different authors have different definitions of BHI. It is therefore easy to become confused over which segment lengthening rate is appropriate. Consequently, we have clearly defined every concept of LG, DI, LL, LI, BHI, and EFI in Table 1 and Fig. 1 to allow better understanding in this study.

In our study, the healing rate and lengthening rate were higher in patients with Ollier’s disease than in those without (shown in Table 3: objective outcomes in both groups), and the difference was especially significant in the femur. In these conditions, the lengthened segment will undergo early consolidation and fail to achieve the target length and angular correction. These findings mean that we should speed up the lengthening rate (DI, LI, BHI, and EFI), increase the range of angular correction according to the patient, shorten the duration of lengthening, and remove the external fixator early to achieve a good therapeutic effect. If done well, the patients could recover well earlier, reducing both the nursing burden and the financial burden.

As to why lower limb lengthening and deformity correction are accelerated in children with Ollier’s disease, Couvineau et al. speculated that tumors initially develop near the growth plate cartilage where endochondral bone ossification occurs. Endochondral ossification is a highly regulated process that requires the differentiation of mesenchymal cells into hypertrophic chondrocytes and the subsequent replacement of the cartilaginous matrix by mineralized bone. It has been postulated that enchondromas result from abnormalities in signaling pathways controlling the proliferation and differentiation of chondrocytes, leading to the development of intraosseous cartilaginous foci [24]. In our study, the lengthening process accelerated in every phase in patients with Ollier’s disease compared to those without. According to the law of tension stress, the lengthening process stimulates cell division, especially in the diseased segment [11], which may influence signaling pathways. However, there is currently no consensus, and the mechanism needs further study.

There were some limitations to this study. First, Ollier’s disease is a rare disease; consequently, this is a small case series and an increase in numbers would improve the validity of the findings. Second, this was a retrospective case series—the data were limited to only what is reported in the medical records and available radiographs. No gait analysis was performed, and we did not evaluate these patients using the pediatric outcomes data collection instrument or visual analog scale. In the future, we will consider using these to measure functional outcomes. Finally, a larger-scale prospective study is warranted, which should be designed to assess the appearance and function of the affected limb in patients with Ollier’s disease.

## Conclusion

Lower-extremity length discrepancy and angular deformity due to Ollier’s disease was corrected effectively by a lengthening technique involving osteotomy and external fixation.

In the femur and tibia, diseased segments had stronger self-repair ability in terms of extension, and the healing speed was obviously faster in patients with Ollier’s disease than in normal bone.

New bone formation accelerated during lower limb lengthening and deformity correction in children with Ollier’s disease.

## Data Availability

We declare that materials described in the manuscript, including all relevant raw data, will be freely available to any scientist wishing to use them for non-commercial purposes, without breaching participant confidentiality.

## Abbreviations

LLD: Length discrepancy
TSF: Taylor spatial frame
CORA: Center of rotation and angulation
AC: Angular correction
LG: Lengthening gap
DI: Distraction index
LL: Lengthening length
L%: Lengthening length percentage
LI: Lengthening index
BHI: Bone healing index
EFI: External fixation index
